# Re-salvage MRI-guided Focal High-dose-rate Brachytherapy for Locally Recurrent Prostate Cancer

**DOI:** 10.7759/cureus.2429

**Published:** 2018-04-05

**Authors:** Marieke J Van Son, Max Peters, Marinus A Moerland, Juus L Noteboom, Wietse S Eppinga, Raquel Davila Fajardo, Jan Lagendijk, Jochem R Van der Voort van Zyp

**Affiliations:** 1 Department of Radiation Oncology, University Medical Center Utrecht; 2 Department of Radiotherapy, University Medical Center Utrecht

**Keywords:** prostate cancer, recurrence, brachytherapy, salvage therapy, interventional magnetic resonance imaging

## Abstract

Prostate cancer recurrences are common, even with twenty-first-century primary prostate cancer treatment modalities. The most common salvage treatment is (delayed) hormonal therapy, which is often associated with serious side-effects. Due to the risk of significant toxicity, whole-gland targeted salvage treatments remain unpopular. Consequently, developments in focal therapies have arisen. Magnetic resonance imaging (MRI)-guided focal salvage high-dose-rate brachytherapy (HDR-BT) is a novel treatment aiming for minimal toxicity in recurrent prostate cancer patients. Repeating focal treatment could, therefore, be possible in case of post-salvage recurrence. We report the case of a 77-year-old man who underwent repeat focal HDR-BT.

## Introduction

Despite improvements in primary prostate cancer care, 15%-55% of patients undergoing radiotherapy develop biochemical recurrence after a 10 years' follow-up [1]. Even with external beam radiation therapy (EBRT) dose escalation, the risk of disease progression remains significant, especially in higher-risk groups [2]. In the management of recurrent disease, physicians are faced with difficult considerations regarding salvage treatment options. Although early recurrences are often confined to the prostate without lymph-node or distant metastases, around 98% of patients are still treated with (delayed) androgen deprivation therapy (ADT). This treatment is associated with significant side-effects, such as erectile dysfunction, osteoporosis, increased risk of diabetes, gynaecomastia, hot flashes, and depression. Moreover, hormonal treatment is palliative and castration resistance usually occurs within one to three years [3]. In contrast, curative whole-gland salvage treatments, such as prostatectomy, brachytherapy, high-intensity focused ultrasound (HIFU), and cryosurgery remain unpopular due to high toxicity rates and, in earlier series, a significant risk of failure [4].

In an effort to reduce toxicity in the salvage setting, research has shifted towards organ-preserving approaches. Although prostate cancer is usually multifocal, the “index lesion” hypothesis states there is one clinically important tumor focus in the prostate (the index lesion), which harbors the metastatic precursor cell [5]. After whole-gland irradiation, the disease often recurs unifocally [6], indicating that smaller secondary lesions have been treated while the index lesion remains. On that premise, salvage focal therapy aimed at this lesion should achieve the same oncological control as whole-gland treatments. The success of focal salvage treatment depends on the degree of tumor visualization and reliable exclusion of metastases. This has significantly improved with advancements in multiparametric (mp)-MRI and prostate-specific membrane antigen (PSMA) positron emission tomography (PET) imaging.

The Radiation Oncology department of the University Medical Center Utrecht (UMCU) is equipped with a 1.5T MRI treatment facility, enabling brachytherapy treatment under MRI guidance. The convergence of these technologies allows for an optimal implantation procedure, supporting focal treatment. In 2013, the UMCU introduced MRI-guided, single-fraction (19Gy) focal high-dose-rate brachytherapy (HDR-BT) with iridium-192 as the salvage treatment for local radio-recurrent prostate cancer. With this treatment, the radiation dose to the tumor is escalated while exposure to the surrounding organs at risk (OAR) is limited. Results with regard to toxicity are promising and, therefore, the question arises whether re-treatment with focal HDR-BT is possible for future post-salvage recurrences. This could prevent or further delay the initiation of ADT, thereby avoiding hormone-induced toxicity. Furthermore, postponing castration resistance could potentially increase prostate cancer-specific survival. We present a novel case of second MRI-guided focal salvage HDR-BT.

## Case presentation

A 77-year-old male visited the radiation oncology department for a follow-up consultation nine years after initial prostate cancer treatment with whole-gland Iodine-125 brachytherapy (145Gy). His further medical history consisted of an asymptomatic thoracic aortic aneurysm and his medication included antihypertensive drugs, a cholesterol-lowering statin, and an anticoagulant. The initial cT1ciT2aNxMx prostate tumor (staged on MRI) was located in the left peripheral zone (initial prostate-specific antigen (PSA) 7.9 ng/ml). Treatment-related toxicity involved increased urinary frequency and transient obstructive complaints for which he received tamsulosin 0.4 mg once daily for approximately 18 months post-implantation. His erectile function declined but was sufficient for penetrative intercourse without the need for supporting medication. PSA levels dropped to a nadir of 0.2 ng/ml one year after treatment and remained stable during the first three years of follow-up. Later, a steady upward trend was seen with a PSA doubling time (PSADT) of 12 months up to the level of 2.5 ng/ml seven years post-treatment. This was considered a biochemical recurrence according to the Phoenix definition (PSA nadir+2 ng/ml) and radiographic evaluation for recurrent disease was performed using 3T multiparametric-MRI (mp-MRI) and F18-Choline PET-CT. However, there were no signs of local recurrence or distant metastases, nor on repeat imaging one year later.

**Figure 1:**
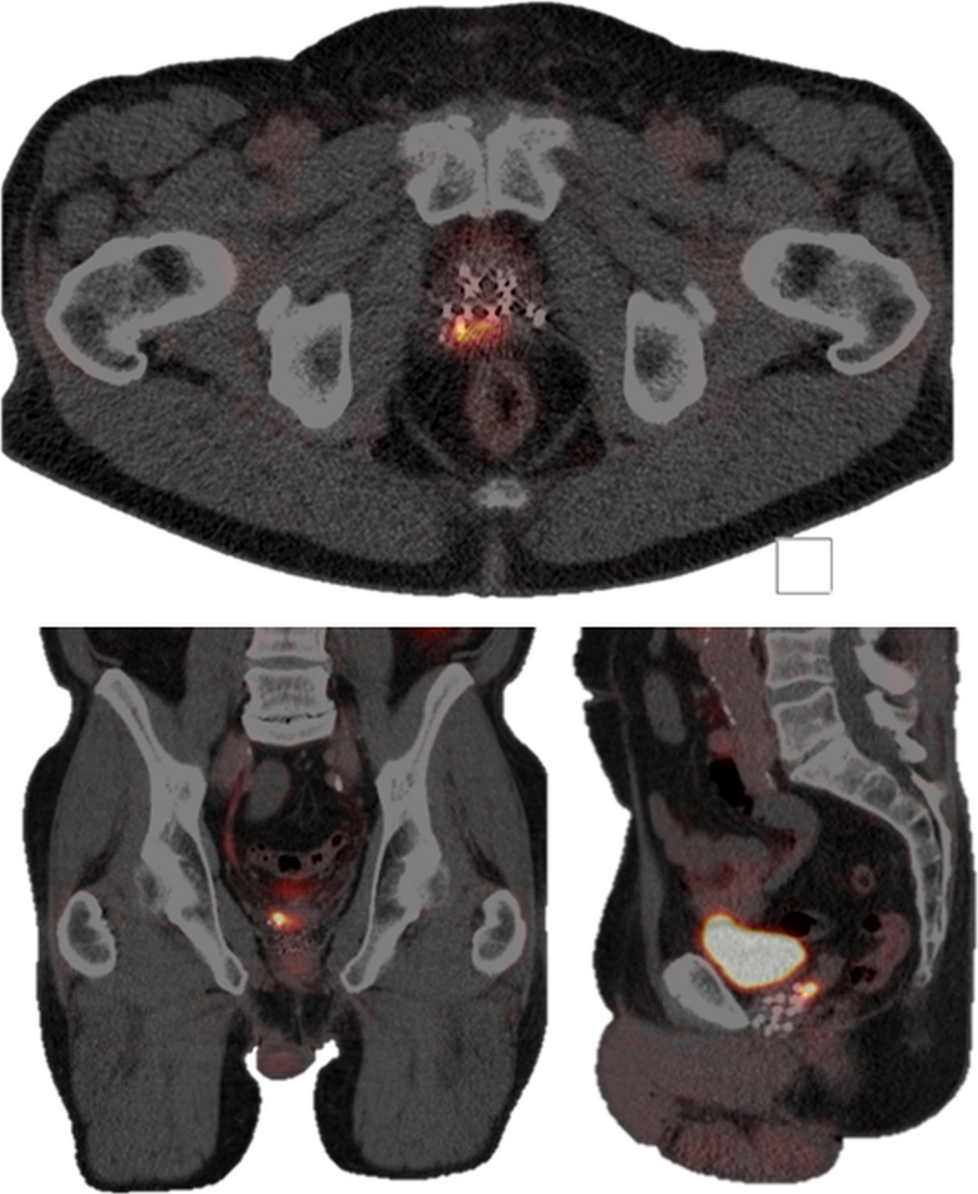
First recurrence lesion on 68Ga-PSMA PET-CT Transversal plane (upper image), coronal plane (lower-left image), and sagittal plane (lower-right image) showing the first recurrence lesion in the right dorsal peripheral zone and in the right seminal vesicle on the 68Ga-PSMA PET-CT, nine years after initial prostate cancer treatment.

**Figure 2:**
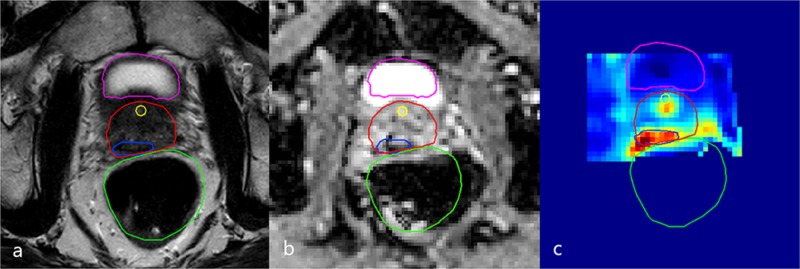
Diagnostic 3T mp-MRI revealing first recurrence lesion Transversal plane of the diagnostic 3T multiparametric-magnetic resonance imaging (mp-MRI) revealing a suspect lesion in the right dorsal peripheral zone next to the prostate midline and in the right seminal vesicle. Delineations of the bladder (purple), urethra (yellow), rectum (green), prostate (red), and gross tumor volume (GTV, blue) are shown. In this case, the clinical target volume (CTV) was considered equal to the GTV because there was mainly seminal vesicle invasion. (a) T2-weighted image, (b) ADC image, (c) K-trans image

When the patient returned to our department, the PSA level had further increased to 6.7 ng/ml (prostate-specific antigen doubling time (PSADT) 18 months) and a 68Ga-PSMA PET-CT followed. The scan revealed local high uptake in the right dorsal peripheral zone next to the prostate midline and in the right seminal vesicle (Figure 1). A 3T mp-MRI (Figure 2) and MRI-guided target biopsies confirmed this lesion (25% adenocarcinoma in one out of two cores, suggested Gleason score 4+3=7). Upon this, the patient was treated with MRI-guided focal salvage high-dose-rate brachytherapy (HDR-BT).

Focal salvage treatment

**Figure 3:**
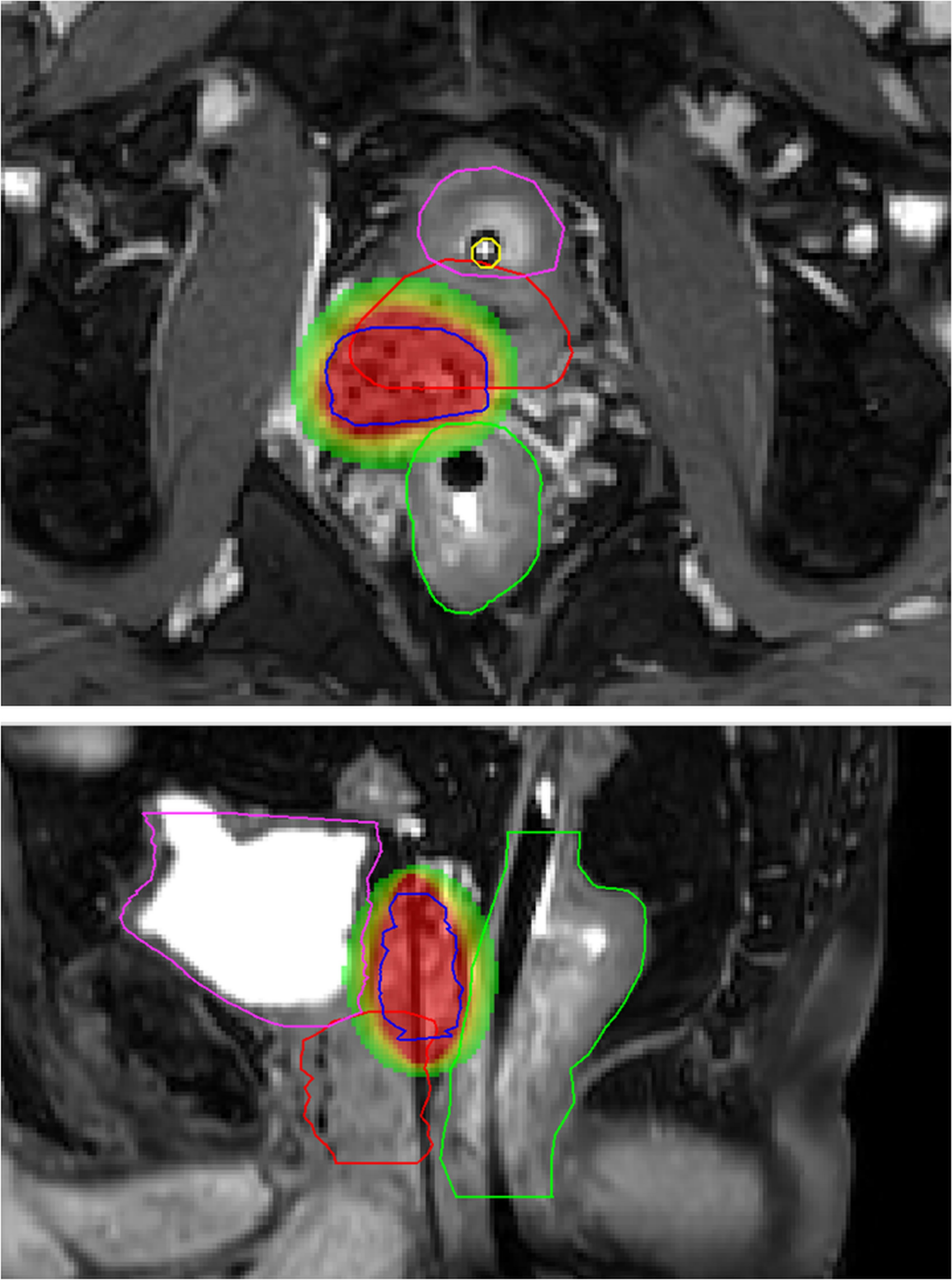
Dose distribution first MRI-guided focal salvage HDR-BT Transversal plane (upper magnetic resonance (MR) image) and sagittal plane (lower MR image) showing delineations of the bladder (purple), urethra (yellow), rectum (green), prostate (red), and gross tumor volume (GTV, blue) in the right dorsal peripheral zone and the right seminal vesicle. The images show signal voids from the catheters and previously implanted iodine 125 seeds. On the sagittal image, one of the catheters is visible within the GTV. In this case, the clinical target volume (CTV) was considered equal to the GTV because there was mainly seminal vesicle invasion. Radiation dose is displayed in colors, with red representing 19Gy and green representing 9.5Gy. MRI: magnetic resonance imaging; HDR-BT: high-dose-rate brachytherapy

Prior to treatment, the gross tumor volume (GTV), clinical target volume (CTV), defined as GTV with a five-millimeter margin, and the organ at risk (OAR) (prostate, bladder, rectum, and urethra) were delineated based on a 3T mp-MRI and 68Ga-PSMA PET-CT scan. For the CTV to planning target volume (PTV), the margin was 0 millimeter as the source and dose distribution along the tumor in brachytherapy. With the patient in the lithotomy position and under spinal anesthesia, seven MR-compatible catheters were placed in and around the right peripheral zone and seminal vesicle via the perineum (Figure 3). Catheter insertion was guided by fused diagnostic MRI delineations and intraoperative transrectal ultrasound. To evaluate catheter positions with respect to the tumor and OAR, an additional 1.5T MRI scan was made and delineations were adjusted to account for swelling. Next, a simulation of dose distribution was made by the treatment planning system. To ensure the safe delivery of the radiation dose, an additional 1.5T MRI scan was made just before the radiation treatment to check for catheter displacements. The dose to 95% of the GTV (D95) was 20.3Gy (aim: >19Gy).The minimum dose to the most exposed 1 cc of rectum or bladder (D1cc) was limited to 10Gy and the dose to 10% of the urethra (D10) was 5.2Gy. We used constraints of D1cc <12Gy for the rectum and bladder and D10 <17.7Gy for the urethra [7]. Later, all catheters were removed, leaving no source of radioactivity behind in the patient. There were no perioperative complications and the patient was discharged the same day.

**Figure 4:**
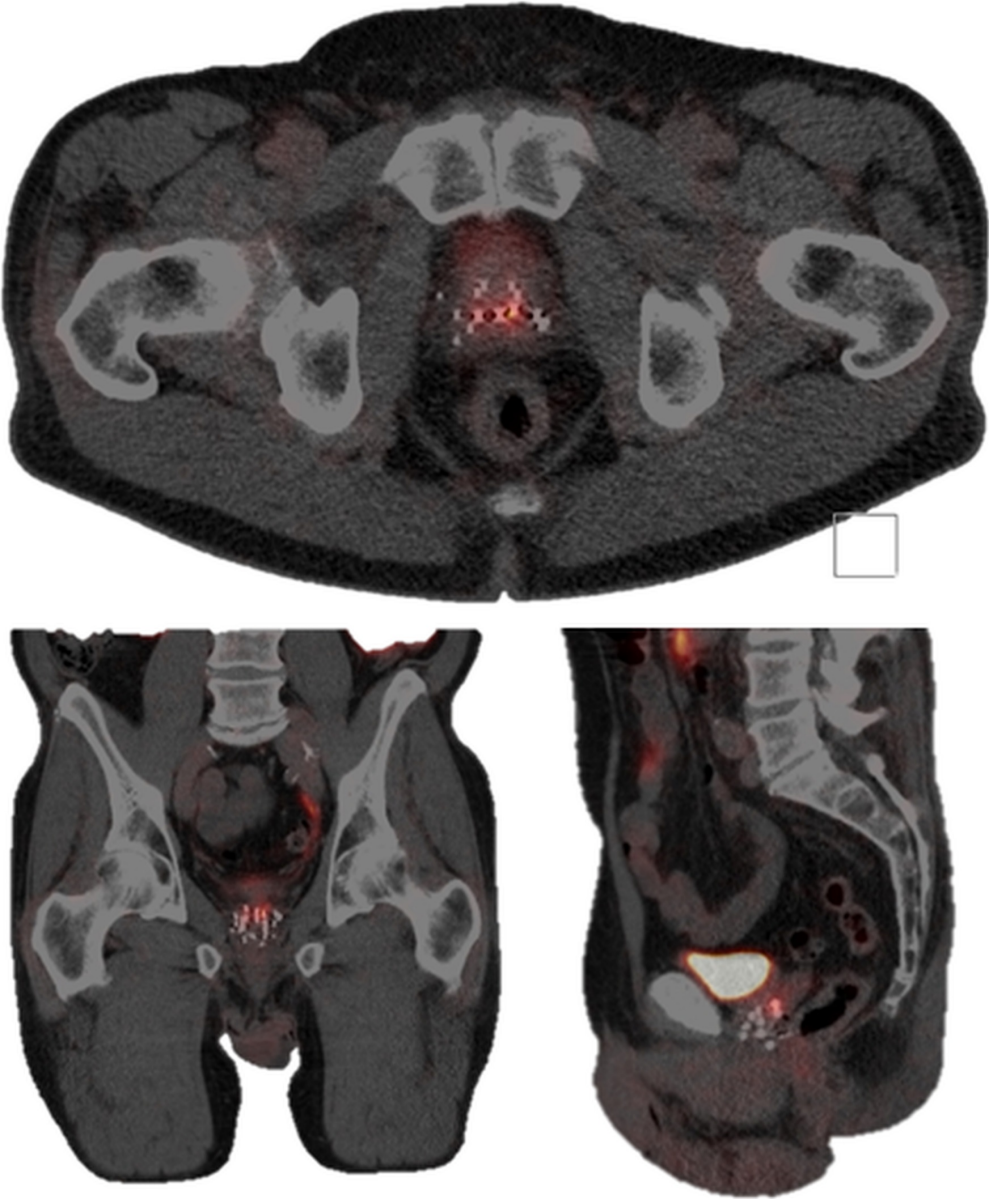
Second recurrence lesion on 68Ga-PSMA PET-CT Transversal plane (upper image), coronal plane (lower-left image), and sagittal plane (lower-right image), showing the second recurrence lesion in the left dorsal peripheral zone on the 68Ga-PSMA PET-CT, 11 years after initial prostate cancer treatment. PSMA PET-CT: prostate-specific membrane antigen positron emission tomography-computed tomography

During the first six months of follow-up, tamsulosin 0.4 mg once daily was prescribed due to minor urinary retention and frequency symptoms. His erectile function decreased in the same period but restored without medication. There were no rectal complaints. Three months post-treatment, the nadir PSA was 0.9 ng/ml. An mp-MRI for response evaluation six months after treatment showed no signs of loco-regional malignant disease and post-radiation fibrosis was visible in the right seminal vesicle. Nevertheless, PSA levels started to rise again, up to 3.4 ng/ml one year after treatment (PSADT, six months). Once again, disease status evaluation was performed with 68Ga-PSMA PET-CT and 3T mp-MRI. As compared to the first diagnostic PET-CT scan, less PSMA uptake was visible in the right peripheral zone. A new suspect lesion of approximately 10 millimeters was suggested in the left dorsal peripheral zone, in close relation to the seminal vesicle (Figure 4). Diminutive diffusion restriction and contrast enhancement on the mp-MRI could not verify this lesion (also due to an overall heterogeneous aspect of the prostate).

**Figure 5:**
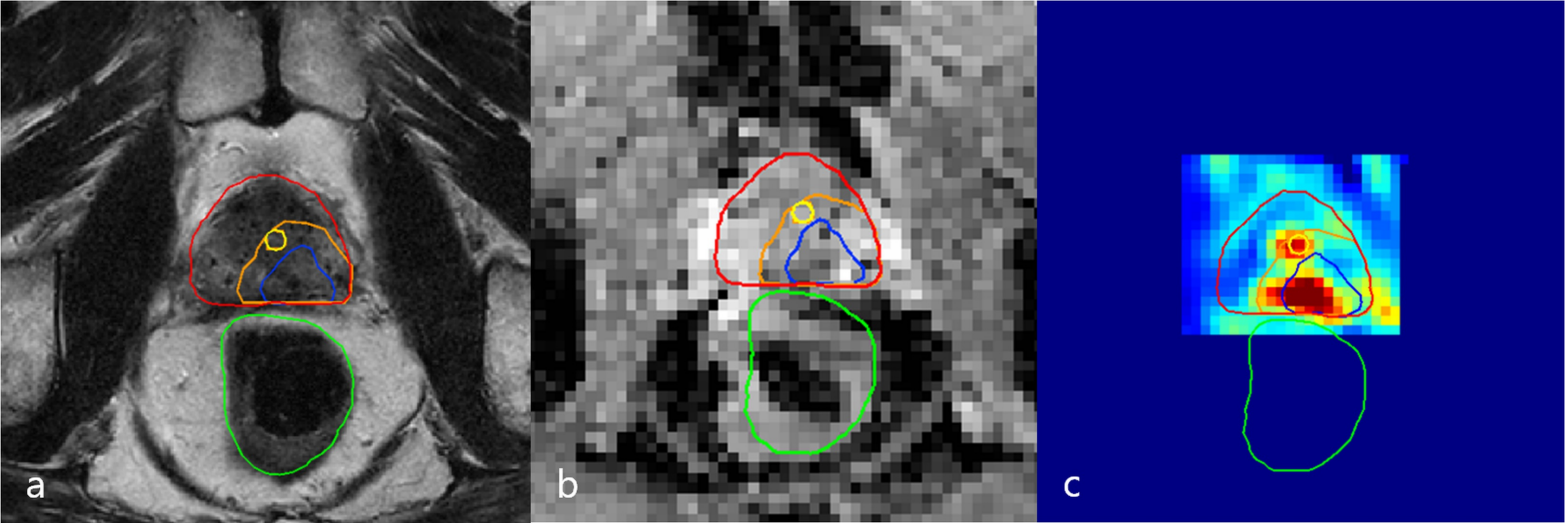
Diagnostic 3T mp-MRI revealing second recurrence lesion Transversal plane of the diagnostic 3T mp-MRI revealing a suspect lesion in the left dorsal peripheral zone. Delineations of the urethra (yellow), rectum (green), prostate (red), gross tumor volume (GTV, blue), and clinical target volume (CTV, orange) are shown. (a) T2-weighted image, (b) ADC image, (c) K-trans image mp-MRI: multiparetic magnetic resonance imaging

Both imaging modalities were repeated six months later at a PSA-value of 4.6 ng/ml (PSADT nine months). The same recurrence location was revealed on the 68Ga-PSMA PET-CT and confirmed by the mp-MRI (Figure 5) and MRI-guided target biopsies (<1% adenocarcinoma in one out of four cores, suggested Gleason score 3+3=6).

Re-salvage treatment

**Figure 6:**
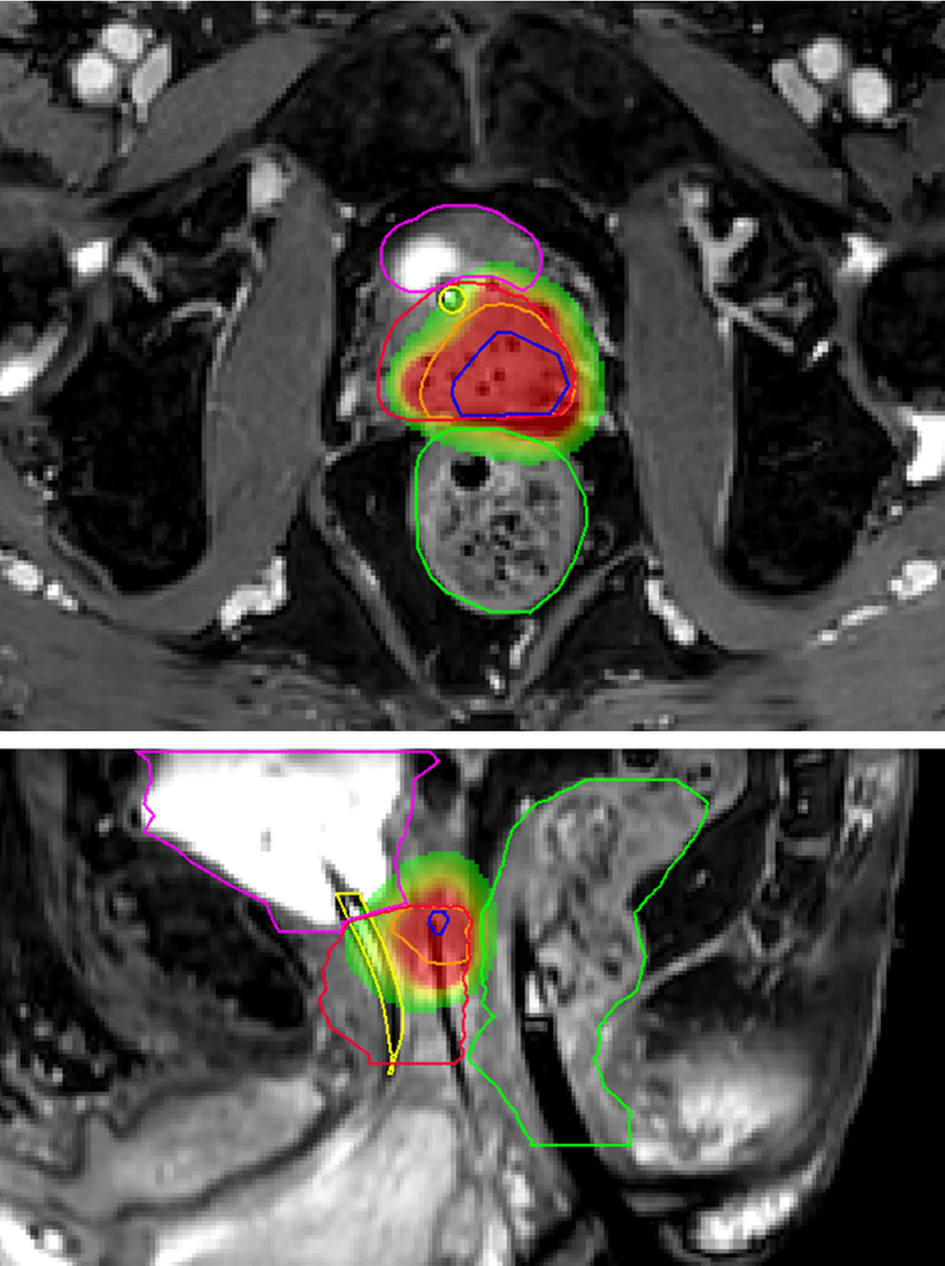
Dose distribution second MRI-guided focal salvage HDR-BT Transversal plane (upper MR image) and sagittal plane (lower MR image) showing delineations of the bladder (purple), urethra (yellow), rectum (green), prostate (red), gross tumor volume (GTV, blue), and clinical target volume (CTV, orange) in the left dorsal peripheral zone. The images show signal voids from the catheters and previously implanted iodine 125 seeds. On the sagittal image, one of the catheters is visible within the CTV. Radiation dose is displayed in colors, with red representing 19Gy and green representing 9.5Gy. HDR-BT: high-dose-rate brachytherapy; MRI: magnetic resonance imaging

**Figure 7:**
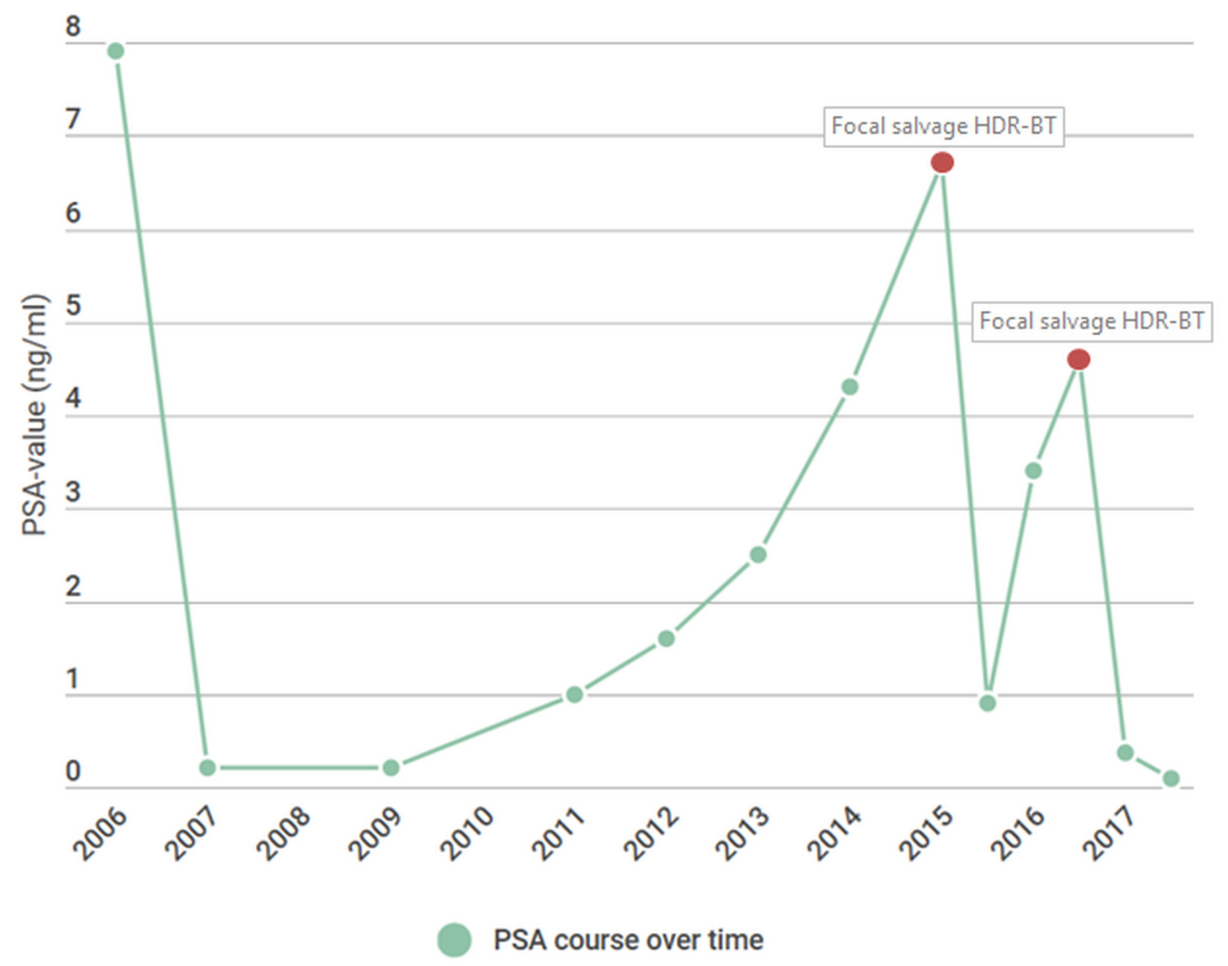
PSA value timeline Timeline showing the course of PSA values over time. The first MRI-guided focal salvage HDR-BT was performed at a PSA level of 6.7 ng/ml. The second MRI-guided focal salvage HDR-BT was performed at a PSA-level of 4.6 ng/ml. HDR-BT: high-dose-rate brachytherapy; MRI: magnetic resonance imaging; PSA: protein-specific antigen

Eleven years after the initial prostate cancer treatment and two years after receiving the first MRI-guided focal salvage HDR-BT, the patient was re-treated. A total of eight catheters were placed in and around the recurrence lesion (Figure 6). D95 for the CTV was 19.1Gy, D1cc of rectum and bladder was 11Gy, and D10 of the urethra was 15.1Gy. No perioperative complications occurred. The postoperative PSA values at one, three, and six months were 0.37 ng/ml, <0.10 ng/ml, and <0.10 ng/ml, respectively. The patient experienced transient flatulence complaints without further need for any therapeutic interventions. Three months after treatment, tamsulosin 0.4 mg was again prescribed. No grade ≥3 toxicity occurred. The mp-MRI six months post-treatment revealed a reduction in both diffusion restriction and contrast enhancement at the treated location. An overview of the course of PSA values over time is presented in Figure 7.

## Discussion

With local salvage treatments, toxicity is a major issue that must be weighed against the benefits of delaying the onset of metastatic disease or, in particular cases, providing a cure. Focal treatment aimed at the tumor lesion instead of the whole prostate gland is a novel development in the span of salvage treatments. Powered by technical advances in diagnostic modalities and the increasing possibilities of MRI guidance before and during treatment, targeted therapy aims to reduce toxicity. With limited toxicity, repetitive salvage treatment could be possible for future local recurrences and ADT (and its side effects) could be postponed or even prevented.

This report describes the first case of repeat MRI-guided focal salvage HDR-BT for prostate cancer recurrence. After both focal treatments, the patient experienced minor toxicity (maximum grade 2), which was limited to urinary retention and frequency symptoms, transient flatulence complaints, and a temporary decrease in erectile function. Because of the relatively short follow-up time after the second treatment, there is no information on long-term toxicity yet. However, results with regard to acute toxicity are promising and major complications in the future are not expected due to the high level of dose control with respect to the OAR. The combination of MRI guidance and the steep dose fall-off in brachytherapy allows for high precision in administering the radiation dose to the tumor. In the described case, dose constraints for the OAR were not exceeded during both focal HDR-BT treatments.

Within the literature, there are few papers covering repeat salvage therapy. One case report by Claren et al. on second salvage treatment using whole-gland HDR-BT (5x7Gy) showed limited toxicity (grade 2 urinary incontinence). After 24 months, a PSA nadir of 0.03 ng/ml was reached [8]. More recently, Maenhout et al. described a case series of four patients receiving MRI-guided focal salvage HDR-BT after previous salvage I-125 brachytherapy. No postoperative development of grade ≥2 toxicity was observed. Lymph node metastatic disease was detected in one patient during follow-up [9].

Choosing the appropriate salvage treatment strategy for recurrent prostate cancer is a complex matter and patient selection for focal treatment depends on many factors. To estimate the risk of toxicity, it is essential to establish any pre-existing urinary or bowel symptoms. Time from treatment to biochemical relapse and PSA kinetics, such as PSADT, have prognostic relevance with respect to salvage oncologic outcomes [10]. Appropriate imaging modalities should be deployed for accurate tumor staging and the detection of disseminated disease. We have adopted 68Ga-PSMA PET-CT and mp-MRI as standard imaging techniques. From our experience, the diagnostic accuracy of this imaging combination has rendered prostate biopsies unnecessary since image-guided biopsies were all tumor-positive in the past. In the salvage setting, the assessment of in- or outfield recurrence is important because infield recurrences with a short interval from the previous treatment (less than two years) may indicate radio-resistancy and are, therefore, less susceptible to repeat focal salvage irradiation.

## Conclusions

MRI-guided focal salvage HDR-BT is a novel modality within the range of local treatment options for recurrent prostate cancer. This case report highlights the potential of this therapy with regard to re-treating locally recurrent prostate cancer after previous salvage treatment. Re-salvage could further delay or even avoid the need for ADT, thereby minimizing the risk of exposure to hormone-related toxicity. The joint use of MRI guidance and HDR-BT allows for targeted therapy with minimal risk of toxicity. Therefore, focal re-treatment seems possible. Further experience in treating patients with re-recurrent local prostate cancer will yield more knowledge on long-term outcomes.
